# Ancient Migratory Events in the Middle East: New Clues from the Y-Chromosome Variation of Modern Iranians

**DOI:** 10.1371/journal.pone.0041252

**Published:** 2012-07-18

**Authors:** Viola Grugni, Vincenza Battaglia, Baharak Hooshiar Kashani, Silvia Parolo, Nadia Al-Zahery, Alessandro Achilli, Anna Olivieri, Francesca Gandini, Massoud Houshmand, Mohammad Hossein Sanati, Antonio Torroni, Ornella Semino

**Affiliations:** 1 Dipartimento di Biologia e Biotecnologie, Università di Pavia, Pavia, Italy; 2 Dipartimento di Biologia Cellulare e Ambientale, Università di Perugia, Perugia, Italy; 3 Department of Medical Genetics, National Institute of Genetic Engineering and Biotechnology, Tehran, Iran; 4 Centro Interdipartimentale “Studi di Genere”, Università di Pavia, Pavia, Italy; University of Cambridge, United Kingdom

## Abstract

Knowledge of high resolution Y-chromosome haplogroup diversification within Iran provides important geographic context regarding the spread and compartmentalization of male lineages in the Middle East and southwestern Asia. At present, the Iranian population is characterized by an extraordinary mix of different ethnic groups speaking a variety of Indo-Iranian, Semitic and Turkic languages. Despite these features, only few studies have investigated the multiethnic components of the Iranian gene pool. In this survey 938 Iranian male DNAs belonging to 15 ethnic groups from 14 Iranian provinces were analyzed for 84 Y-chromosome biallelic markers and 10 STRs. The results show an autochthonous but non-homogeneous ancient background mainly composed by J2a sub-clades with different external contributions. The phylogeography of the main haplogroups allowed identifying post-glacial and Neolithic expansions toward western Eurasia but also recent movements towards the Iranian region from western Eurasia (R1b-L23), Central Asia (Q-M25), Asia Minor (J2a-M92) and southern Mesopotamia (J1-Page08). In spite of the presence of important geographic barriers (Zagros and Alborz mountain ranges, and the Dasht-e Kavir and Dash-e Lut deserts) which may have limited gene flow, AMOVA analysis revealed that language, in addition to geography, has played an important role in shaping the nowadays Iranian gene pool. Overall, this study provides a portrait of the Y-chromosomal variation in Iran, useful for depicting a more comprehensive history of the peoples of this area as well as for reconstructing ancient migration routes. In addition, our results evidence the important role of the Iranian plateau as source and recipient of gene flow between culturally and genetically distinct populations.

## Introduction

The Middle Eastern region had a central role in human evolution. It has been a passageway for *Homo sapiens* between Africa and the rest of Asia and, in particular, the first region of the Asian continent occupied by modern humans [1]–[Bibr pone.0041252-Stringer1] [3]. This area was also one of the regions where agriculture began during the Neolithic period, in particular in the Fertile Crescent, from which it spread westwards and eastwards. Different pre-historic sites across the Iranian plateau point to the existence of ancient cultures and urban settlements in the sixth millennium BP, perhaps even some centuries earlier than the earliest civilizations in nearby Mesopotamia [4]. Proto-Iranian language first emerged following the separation of the Indo-Iranian branch from the Indo-European language family [5]. Proto-Iranians tribes from Central Asian steppes arrived in the Iranian plateau in the fifth and fourth millennium BP, settled as nomads and further separated in different groups. By the third millennium BP, Cimmerians, Sarmatians and Alans populated the steppes North of the Black Sea, while Medes, Persians, Bactrians and Parthians occupied the western part of the Iranian plateau. Other tribes began to settle on the eastern edge, as far East as on the mountainous frontier of north-western Indian subcontinent and into the area which is now Baluchistan. The nowadays Iranian territory had been occupied by Medes (Maad) in the central and north-western regions, Persians (Paars) in the south-western region and by Parthians (Parthav) in the north-eastern and eastern regions of the country. In the 6^th^ century BC Cyrus the Great founded the Achaemenid Empire (the first Persian Empire), which started in South Iran and spread from Libya to Anatolia and Macedonia, encompassing an extraordinary ethno-cultural diversity [6]. This widespread empire collapsed after two centuries (towards the end of the 4^th^ century BC) on account of Alexander the Great. In the 2^nd^ century BC, north-eastern Persia was invaded by the Parthians who founded an empire extending from the Euphrates to Afghanistan. Because of its location on the Silk Road, connecting the Roman Empire and the Han Dynasty in China, it quickly became a centre of trade and commerce. The Parthians were succeeded by the Sassanid Empire, one of the most important and influential historical periods of Persia. Afterwards Iran was invaded by several populations such as the Arabs, Mongols and Ottoman Turks. The Muslim conquest of Persia in 637 AC led to the introduction of Islam, with the consequent decline of the Zoroastrian religion [7], which still survives in some communities in different part of Iran, especially in Tehran and Yazd.

This continuous invasion of populations with different origin and culture created an interesting mix of different ethnic groups speaking a variety of Indo-Iranian, Semitic and Turkic languages and encompassing Arabs, Armenians, Assyrians, Azeris, Baluchs, Bandaris, Gilaks, Kurds, Lurs, Mazandarani, Persians, Qeshm people, Turkmens, Zoroastrians and a group of so-called Afro-Iranians, which might be the result of the slave trade with Zanzibar. Despite the great potentiality of this genetic scenario in providing useful information to reconstruct traces of ancient migrations, only few studies have investigated the multi-ethnic components of the Iranian gene pool [8]–[Bibr pone.0041252-Nasidze1] [Bibr pone.0041252-Farjadian1] [Bibr pone.0041252-Nasidze2] [Bibr pone.0041252-Regueiro1] [Bibr pone.0041252-Farjadian2] [Bibr pone.0041252-Lashgary1] [15].

In order to shed some light on the genetic structure of the Iranian population as well as on the expansion patterns and population movements which affected this region, the Y-chromosomes of 938 Iranians, representative of the majority of the provinces and ethnic groups in Iran, were examined at an unprecedented level of resolution.

### Major Iranian ethnic groups

*Arab-speakers* in Iran are mainly scattered along the Persian Gulf coast. The main unifying feature of this group is a Semitic language, “the Arabic”, originated in the Arabian Desert from where it diffused among a variety of different peoples across most of South-West Asia and North Africa determining their acculturation and eventual denomination as Arabs. As in most cases, their presence in Iran is due to the process of Islamization of Persia started in the 7^th^ century that led to the decline of the Zoroastrian religion. Although after the Arab invasion many Arab tribes settled in different parts of Iran, at present they are the main ethnic group of Khuzestan, where they have maintained their identity probably also for a continuous influx of Arab-speaking immigrants into the province from the 16^th^ to the 19^th^ century.

*Armenians* are descendants of people with Armenian origin. Armenia historically corresponded to a region characterized by three lakes now divided among Turkey, Iraq and Iran countries, once part of the Hittite Empire. With the conquest of Alexander the Great, Armenia became part of the Macedonian Empire coming into contact with European civilization. Armenians arrived into Iran in 1600 as captives and the present-day community is a Christian minority of no more than 100,000 individuals who mostly live in Tehran and the Jolfa district of Isfahan [14].

*Assyrians* are Semitic people speaking Aramaic dialects and represent the second Christian community in Iran. They live mainly in Azerbaijan Gharbi; the community present in Tehran originated at the beginning of the last century with the return of Assyrian refugees from Iraq where they fled during the First World War [16]. Although at present they represent an Iranian minority, during the Assyrian Empire (911–608 BC) they played an important role controlling much of the western part of the Iranian country (including Media, Persia, Elam and Gutium). Their ancestors are among the oldest Middle Eastern groups with origin in the Fertile Crescent and the principal promoters of the development of Mesopotamian civilization. During their regime, conquered peoples were moved inside the empire, acculturated and then assimilated as loyal components making the Assyrian Empire a multi-ethnic state. With the fall of the Assyrian Empire in 539 BC and the coming into power of the Persians, Assyrians remained in north-western Iran for many thousands of years where, as Armenians, for their religious and cultural traditions, had little intermixture with the other groups: Assyrians and Armenians are thus good representatives of ancient Middle Eastern populations.

*Azeris* are mainly Shi'a Muslims and are the largest ethnic group in Iran after the Persians. The name “Azeri” is a Turkified form of “Azari” and the latter is derived from the Old Iranian name for the region of Azerbaijan in North-West Iran. The Azari people likely derive from ancient Iranic tribes, such as the Medians in Iranian Azerbaijan. Azari was the dominant language there before it was replaced in many regions by the Turkic language. It was spoken in most of Azerbaijan at least up to the 17th century, with the number of speakers decreasing since the 11th century due to the Turkification of the area. During the time of the Mongol invasion, most of the invading armies were composed of Turkic tribes, which increased the influence of Turkish in the region. Today, the Azari language is completely replaced by Turkish or Azeri language. The question remains whether this language replacement happened with Turkish people gene flow or it happened simply as a result of acculturation without gene flow.

*Baluchis* live in Sistan and Baluchestan (a province of South-East Iran) but also in Afghanistan, Oman and Pakistan. They are Sunni Muslims, in contrast to the Sistani Persians who are adherents of Shia Islam. Although their origin is still unknown, it seems that this group is likely descendant of ancient Median and Persian tribes coming from the Caspian Sea and first settled in northern Persia.

*Gilaks and Mazandarani*, also called Caspian people, are closely related. They live in North Iran although they are thought to have originated from the South Caucasus. Gilaks and Manzandarani are part of the northern branch of the western Iranian languages and are closely related, even if they share also many common words with Persian and Kurdish, belonging to different Iranian language branches.

*Kurds* are considered an ethnic group since the medieval period. The prehistory of the Kurds is poorly known, but their ancestors seem to have inhabited the same inhospitable mountainous region for millennia remaining relatively unmixed with the invaders. The records of the early empires of Mesopotamia contain frequent references to mountain tribes with names resembling “Kurd”. They inhabit broad lands from the Azerbaijan to Khuzestan but in the 17^th^ century a large number of Kurds were also present in Khorasan.

*Lurs* are one of the major Iranian ethnic groups inhabiting along the central and southern parts of the Zagros Mountains. Their origin might go back to the time before the migration of Indo-Europeans to Iran when other groups called Elamites and Kassites were living there [17]. The Kassites are said to be the native people of Lorestan and their language was neither Semitic nor Indo-European and differed from the Elamite. The modern Lurs, like the Kurds, are a mixture of these aboriginal groups and invading Indo-Iranians from which it is thought they separated. Until the 20^th^ century, the majority of Lurs were nomadic herders. Recently, the vast majority of Lurs have settled in urban areas although a number of nomadic Lur tribes still persist.

*Persian* identity refers to the Indo-European Aryans who arrived in Iran about 4 thousand years ago (kya). Originally they were nomadic, pastoral people inhabiting the western Iranian plateau. From the province of Fars they spread their language and culture to the other parts of the Iranian plateau absorbing local Iranian and non-Iranian groups. This process of assimilation continued also during the Greek, Mongol, Turkish and Arab invasions. Ancient Persian people were firstly characterized by the Zoroastrianism. After the Islamization, Shi'a became the main doctrine of all Iranian people.

*Turkmen* came from the Altai Mountains in the 7^th^ century AC, through the Siberian steppes. They now live in Golestan and are different from the other ethnic groups in appearance, language and culture.

*Zoroastrians* are the oldest religious community in Iran; in fact the first followers have been the proto-Indo-Iranians. With the Islamic invasions they were persecuted and now exist as a minority in Iran.

## Materials and Methods

### The sample

The sample consisted of 938 unrelated males from 14 Iranian provinces and belonging to 15 different ethnic groups (in parentheses): 102 from Azerbaijan Gharbi (39 Assyrians, 63 Azeris), 44 from Fars (Persians), 64 from Gilan (Gilaks), 68 from Golestan (Turkmens), 192 from Hormozgan (131 Bandari, 49 Qeshmi, 12 Afro-Iranians), 11 from Isfahan (Persians), 59 from Khorasan (Persians), 57 from Khuzestan (Arabs), 59 from Kurdistan (Kurd), 50 from Lorestan (Lurs), 72 from Mazandaran (Mazandarani), 24 from Sistan Baluchestan (Baluchs), 56 from Tehran (34 Armenians, 9 Assyrians, 13 Zoroastrians), 80 from Yazd (46 Persians, 34 Zoroastrians). Geographical and ethnological information such as ethnicity, language and genealogy were ascertained by interview after having obtained their informed consent. DNA was extracted from whole blood by using standard phenol/chloroform protocol.

### Ethics Statement

This research has been approved by the Ethic Committee for Clinical Experimentation of the University of Pavia, Board minutes of the 5^th^ of October 2010. Geographical and ethnological information such as ethnicity, language and genealogy were ascertained by interview after having obtained their written informed consent.

### Population samples employed for comparisons

Population samples from the following neighbouring countries/regions were used for comparison: Ethiopian Amhara (ETA, N = 48), Ethiopian Oromo (ETO, N = 78) [18]; Iraqi from Baghdad (IRQ, N = 154) [19], [20]; Sardinian (SARD, N = 520) [21]; Tunisian (TUN, N = 148) [22]; Central Turkish (C-TK, N = 152), Eastern Turkish (E-TK, N = 208), Western Turkish (W-TK, N = 163) [23]; Arab from Egypt (EG-A, N = 147), Omani (OMA, N = 121) [3]; Austro-Asiatic Indian (IND-AA, N = 64), Dravidian Indian (IND-D, N = 353), Indo-European Indian (IND-IE, N = 224), Tibeto-Burman Indian (IND-TB, N = 87), Burushaski Pakistani (PAK-B, N = 20), Dravidian Pakistani (PAK-D, N = 25), Indo-European Pakistani (PAK-IE, N = 132) [24]; United Arab Emirates (UAE, N = 164), Yemeni (YEM, N = 62), Qatari (QAT, N = 72) [25]; Saudi Arabian (SAR, N = 157) [26]; Albanian and Former Yugoslavia Republic of Macedonia Albanian (ALB, N = 119), Balkarian (BK, N = 38), Bosnia-Erzegovinian (BOS, N = 255) Croatian (CRO, N = 118), Czech (CZE, N = 75), Georgian (GEO, N = 66), Greek (GRE, N = 149), Hungarian (HUG, N = 53), North-East Italian (NEI, N = 67), Polish (POL, N = 99), Slovenian (SLV, N = 75), Ukrainian (UKR, N = 92) [27]; Iraqi Marsh Arab (IRM, N = 143) [20]; South-West Altaian (SW-ALT, N = 30), South-East Altaian (SE-ALT, N = 89) [28]; North Afghanistan (N-AF, N = 44), South Afghanistan (S-AF, N = 146) [29].

### Molecular analysis

Eighty-eight Y-chromosome binary genetic markers were hierarchically genotyped as AFLP (YAP, [30]), RFLP (M2 [31], SRY_10831.2_ [32], M12 [33]; P15 [34]; M74 [35]; M34, M60, M61, M67, M70, M76, M78, M81, M175, M198, M207, M213 [36]; LLY22g, P36.2, P43 [37]; M123, M172 [38]; M242, M253, M285 [23]; V12, V13, V22 [39]; M377 [24]; P128, P287 [40]; M406 [41]; M269 [42]; Page08 [43]; V88 [44]; M458 [45]; PAGE55 [46]; L23, M412 [47]; L91 [48]; M527, M547, Page19, P303, U1 [49]), by DHPLC (M217 [50]; M25, M35, M47, M68, M69, M82, M92, M124, M170, M173, M174, M201, M205, M214, M216 [36]; M429 [51]; P209 [40]; M241, M267, M343 [23]; M357, M378, M410 [24]; M346 [40]; M434, M458 [45]; M530 [46]; L497, P16 [49]), and direct sequencing (M18 [33]; M42, M73, M75, M96 [52]; M33, PN2 [36]; MEH2 [53]; M317 [24]; M356 [54]; M438 [51]; P297 [40]).

The nomenclature used for haplogroup labeling is in agreement with the YCC conventions [37] and recent updates [24], [40], [43], [Bibr pone.0041252-Underhill3] [Bibr pone.0041252-King2] [45]–[Bibr pone.0041252-King2] [47], [49], [51].

The following 10 Y-STR loci: DYS19, DYS388, DYS389I/II, DYS390, DYS391, DYS392, DYS393, DYS439, DYS460, YCAIIb/YCAIIa were analyzed in a subset of Y chromosomes belonging to the most represented haplogroups in the population, by using a 3730 Applied Biosystems sequencer as previously described [27].

### Statistical analyses

Haplogroup diversity was computed using the standard method of Nei [55]. Comparison between groups was performed using the Chi Square Test of independence (StatView package). Genetic structure was examined through the Analysis of MOlecular VAriance (AMOVA [56]) using the Arlequin software Ver 3.5, adopting different grouping criteria (geographic, ethnic, linguistic and religious). Two parallel tests were carried out: one, at a low resolution level, including all compared populations listed above; the other, restricted to the Iranian population samples, at the resolution level reached in this survey. Principal Component Analysis (PCA) on haplogroup frequencies (Table S1, disregarding those lower than 5%) was conducted with Excel, through Xlstat add-in. Within specific haplogroups, Median-Joining (MJ) networks [57] were constructed using Network 4.6.0.0 program (Fluxus Engineering, http://www.fluxus.engineering.com), after having processed data with the reduced-median method [58] and weighted the STR loci proportionally to the inverse of the repeat variance. Geographical view of the haplogroup frequency and mean variance distributions were obtained by using Surfer 6.0 (Golden Software) following the Kriging procedure, as previously described [27]. The maps of microsatellite variances were obtained after having pooled data from locations with less than 5 observations and assigned the resulting values to the centroid of the pooled locations. The age of microsatellite variation was evaluated using the method proposed by Zhivotovsky et al. [59] and modified according to Sengupta et al. [24].

## Results and Discussion

### Structure of the Y-chromosome gene pool in Iran

**Figure 1:**
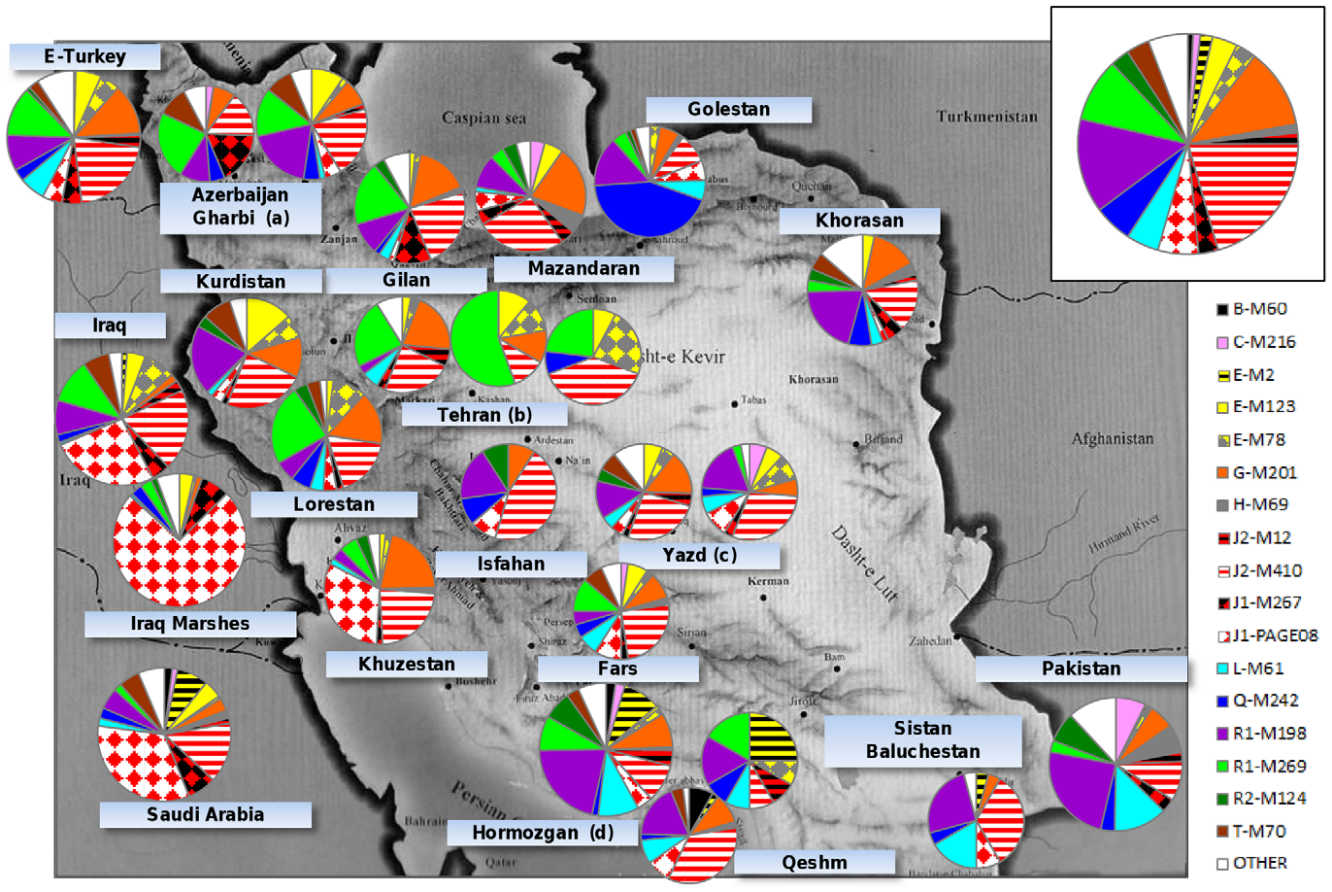
Frequencies of the main Y-chromosome haplogroups in the whole Iranian population (inset pie), in the 14 Iranian provinces under study and in East Turkey [**23**], Iraq [**20**], Saudi Arabia [**26**] and Pakistan [**24**]). (a) Azeris and Assyrians, (b) Armenians, Assyrians and Zoroastrians, (c) Persians and Zoroastrians, (d) Bandari and Afro-Iranians. Pie areas are proportional to the population sample size (small pies, N<50; intermediate pies, 50<N<100; large pies, N>100) and the areas of the sectors are proportional to the haplogroup frequencies in the relative population.

The analysis of 88 Y-chromosome bi-allelic markers in 938 subjects belonging to 15 ethnic groups from 14 Iranian provinces allowed the identification of 65 different Y-chromosome lineages (Table 1 and Figure S1). They belong to 15 main haplogroups (B, C, D, E, F, G, H, I, J, L, N, O, Q, R and T) the most frequent of which are J (31.4%), R (29.1%), G (11.8%) and E (9.2%), with great differences (disregarding those relative to samples smaller than 20 subjects) in frequencies and sub-haplogroups observed among provinces and ethnic groups (Figure 1).

**Table 1:** Haplogroup frequencies (%) in the examined Iranian groups.

	PROVINCE	IRAN	AZARBAIJANGHARBI		FARS	GILAN	GOLESTAN	HORMOZGAN			ISFAHAN	KHORASAN	KHUZESTAN	KURDESTAN	LORESTAN	MAZANDARAN	SISTANBALOUCHESTAN	TEHRAN			YAZD	
Haplogroup	Ethnic group		Assyrian	Azeri	Persian	Gilak	Turkmen	Bandari	Gheshmi	Afro-Iranian	Persian	Persian	Arab	Kurd	Lur	Mazandarani	Baluch	Armenian	Assyrian	Zoroasterian	Persian	Zoroasterian
	**N**	**938**	**39**	**63**	**44**	**64**	**68**	**131**	**49**	**12**	**11**	**59**	**57**	**59**	**50**	**72**	**24**	**34**	**9**	**13**	**46**	**34**
B	M60	**0.7**						2.3	8.2													
C*	M216*	**0.1**																				2.9
C3	M217	**0.4**	2.6					0.8								1.4						2.9
C5	M356	**0.5**			2.3			1.5								2.8						
E1b1a1	M2	**1.8**						9.2	2.0	25.0							4.2					
E1b1b1	M35*	**0.3**			2.3		1.4	0.8														
E1b1b1a1	M123*	**0.2**											1.8			1.4						
E1b1b1a1a	M34	**3.5**		9.5	6.8	1.6		0.8				3.4		13.6	2.0	4.2		2.9	11.1	7.7	6.4	5.9
E1b1b1a1b	M78*	**1.3**					2.9	1.5						1.7	9.8			2.9		7.7		
E1b1b1a1c	V12	**0.1**				1.6																
E1b1b1c	V13	**1.1**		1.6						8.3				5.1					11.1		4.3	5.9
E1b1b1c1	V22	**0.5**			2.3								1.8							15.4		2.9
E2	M75	**0.3**						2.3														
F*	M89*	**0.1**						0.8														
G1	M285	**1.8**	5.1		2.3	1.6		3.1				1.7		3.4	2.0	4.2	4.2	2.9				
G2*	P287*	**0.9**		1.6					2.0				1.8	3.4				5.9			2.1	
G2a*	P15*	**3.5**	2.6	3.2		3.1	1.4	2.3	2.0		9.1	3.4	10.5	5.1	3.9	4.2		5.9			4.3	5.9
G2a	L91	**0.1**			2.3																	
G2a1	P16	**0.2**				1.6		0.8														
G2a3	M406	**0.7**			2.3		1.4	0.8				1.7	1.8								4.3	
G2a3a	PAGE19	**0.1**							2.0													
G2a3a1	M547*	**0.4**						1.5					3.5									
G2a3b*	P303*	**3.3**		1.6	2.3	9.4	2.9					6.8	3.5		9.8	11.1		2.9			2.1	
G2a3b2	U1	**0.4**														1.4		2.9	11.1		2.1	
G2a3b2a	M527	**0.2**							4.1													
G2c	M377	**0.1**		1.6																		
H*	M69*	**0.1**														1.4						
H1a	M82	**1.2**			2.3	1.6	1.4	0.8	2.0			3.4	1.8			4.2						
IJ	M429*	**0.2**			2.3											1.4						
I1	M253	**0.2**				1.6												2.9				
I2	M438	**0.3**						0.8						1.7				2.9				
J1	M267*	**2.9**	17.9		2.3	10.9						5.1	1.8	1.7	2.0	4.2		2.9			2.1	2.9
J1c3	PAGE08	**6.0**		4.8	9.1	1.6	5.8	3.8	8.2		9.1	1.7	31.6	3.4	3.9	5.6	8.3				4.3	8.8
J2*	M172*	**0.1**											1.8									
J2a*	M410*	**2.8**		4.8		6.3		0.8			27.3	6.8	7.0	1.7	2.0	6.9						
J2a3*	PAGE55*	**4.8**	10.3	4.8	9.1	4.7	4.3	2.3	10.2	8.3		1.7	3.5	5.1	2.0	1.4	12.5	2.9		23.1	6.4	2.9
J2a3a	M47	**3.5**		4.8	6.8	3.1	1.4		6.1			3.4	7.0	1.7	5.9	6.9		5.9			2.1	8.8
J2a3b*	M67*	**2.6**	2.6	3.2	4.5	4.7		1.5	6.1		9.1			6.8	3.9	1.4		8.8				
J2a3b1	M92	**1.0**					1.4	1.5			9.1		1.8	1.7			12.5					
J2a3h	M530	**6.1**	2.6	3.2	4.5	4.7	1.4	3.1	12.2			3.4	3.5	6.8	3.9	9.7	8.3	5.9	11.1	15.4	17.0	17.6
J2b1	M205	**0.9**						2.3				1.7				2.8		5.9				
J2b2	M241	**0.7**		1.6				1.5		8.3						1.4					4.3	
L*	M61*	**0.2**		1.6		1.6																
L1	M76	**1.8**			2.3	1.6		5.3	8.2					1.7			8.3				2.1	
L2	M317	**1.5**						4.6		8.3			1.8		3.9	1.4		2.9				5.9
L3	M357	**1.5**		1.6	4.5	1.6	5.8					3.4					8.3	2.9			2.1	
NO*	LLY22g*	**0.3**			2.3		2.9															
N	M214	**0.1**		1.6																		
O	M175	**0.3**										3.4										2.9
Q1*	P36.2*	**0.3**	2.6									1.7								7.7		
Q1a1	M120	**0.1**															4.2					
Q1a2	M25	**3.2**		1.6			42.6															
Q1a3	M346	**0.9**		1.6	4.5	1.6		0.8		8.3					2.0							2.9
Q1b1	M378	**1.1**	2.6	1.6				0.8	2.0		9.1	5.1			3.9							
R*	M207*	**0.6**	2.6					0.8				3.4				1.4		2.9				
R1*	M173*	**1.2**	5.1			3.1		0.8				1.7	1.8	1.7			4.2				4.3	
R1a*	SRY1532*	**0.4**						0.8	2.0			1.7				1.4						
R1a1a*	M198*	**13.9**	10.3	19.0	4.5	9.4	14.5	21.4	18.4	16.7	18.2	20.3	3.5	20.3	5.9	9.7	25.0	2.9			12.8	17.6
R1b*	M343*	**0.7**		3.2		1.6						1.7		1.7							4.3	
R1b1a1	M73	**0.1**										1.7										
R1b1a2*	M269*	**0.5**		1.6				0.8								1.4				15.4		
R1b1a2a*	L23*	**8.5**	23.1	12.7	11.4	18.8	4.3	6.1		16.7		3.4	3.5		23.5	2.8		23.5	55.6	7.7		2.9
R1b1a2a1a	M412	**0.3**						1.5					1.8									
R2	M124	**2.8**				3.1	1.4	6.9			9.1	3.4	3.5	3.4	3.9	4.2					4.3	
T	M70	**3.4**	10.3	7.9	6.8		1.4	3.1	4.1			5.1		8.5	3.9						6.4	
	**Diversity** (a)	**.952**	.896	.931	.962	.933	.793	.930	.926			.943	.883	.925	.918	.954	.909	.936			.942	.929

a Haplogroup diversity provided only for samples with size larger than 20.

On the whole, the Iranian population is characterized by very high haplogroup diversity (0.952): the maximum value being observed in the Persians of Fars (0.962) and the minimum in the Arabs of Khuzestan (0.883) and the Turkmen of Golestan (0.821).

**Haplogroup J** is predominant in Iran where both its sub-clades, J2-M172 and J1-M267, are observed. Its highest frequencies are registered in the populations located along the south-western shores of the Caspian Sea and along the Zagros Mountains ridge. Exceptionally high is the frequency observed in the Baluchi of Sistan Baluchestan, in agreement with their likely Caspian Sea origin.

J1-M267 does not exceed 10% in the majority of the Iranian samples examined, with higher values only in Fars (11.4%), Zoroastrians from Yazd (11.7%), Gilan (12.5%), Assyrians from Azerbaijan (17.9%) and Khuzestan (33.4%). The proportion of the two sub-lineages, J1-Page08 and J1-M267*, is highly variant, being J1-M267* almost restricted to north-western Iranian groups and J1-Page08 mainly observed in populations living below the Dasht-e Kevir and Dasht-e Lut desert area, (approximately latitude 30°N). It reaches a frequency of 31.6% in the Arab group from Khuzestan at the border with southern Iraq.

J2-M172 is the main Iranian haplogroup (22.5%), almost entirely (92.9%) represented by J2a-M410 sub-clades.

The majority of the M410 chromosomes are J2a-Page55 and mainly represented by its main sub-clades M530, M47 and M67. In particular, the recently described J2a-M530 [46] shows high frequencies in the Zoroastrians of Yazd (17.6%) and Tehran (15.4%), and in the Persians of Yazd (17.0%). J2a-M47 reaches frequencies higher than 5% in the Zoroastrians of Yazd (8.8%), in Mazandaran, Khuzestan and Fars (∼7%), while it is absent in the Assyrians of Azerbaijan Gharbi and Tehran, in Sistan Baluchestan and in Hormozgan (except for the Qeshm group). J2a-M92 was observed in Sistan Baluchestan (12.5%) while the paragroup J2a-M67* was observed mainly in the Armenians of Tehran (8.8%). J2a-M68, previously reported in the neighbouring Iraqi population [20], [60], was not observed in Iran. As for the paragroups, J2a-M410* represents 2.8% of the total sample with ∼7% of frequency in Khuzestan, Mazandaran and Khorasan, whereas J2a-Page55*, observed at 6.6% in central Anatolia [46], accounts for 4.8% of the Iranian sample. J2-M172*, recently described in the neighbouring Iraqi Marsh Arabs (3.5%) [20], characterizes one subject from Khuzestan (1.8%).

**Haplogroup R** in Iran is mainly represented by the R1 sub-lineages R1a-M198 and R1b-M269, whereas R2-M124 was observed only in 2.8% of the total sample. All the R1a Y chromosomes belong to the M198* paragroup with frequencies ranging from 0% to 25%. Indeed neither the “European” M458 nor the “Pakistani” M434 [45] have been observed in our samples. Haplogroup R1b-M269 shows its highest frequency in the Assyrians (29.2%, averaged on Tehran and Azerbaijan Gharbi groups). High values are also observed in the Armenians from Tehran and in Lorestan (both with ∼24%). With the exception of five chromosomes belonging to the paragroup R1b-M269* and three chromosomes clustering in the “European” sub-haplogroup R1b-M412, all the M269 Y chromosomes belong to the R1b-L23 clade.

**Haplogroup G** is observed in this survey as G1-M285 and G2a-P15. G1-M285, previously described in the Iranian population [12], accounts only for 1.8% of the present Iranian sample. G2a-P15 is the most frequent sub-clade characterizing 9.1% of the total sample, with incidences ranging from 0% in Sistan Baluchestan to 19.3% in the Arabs of Khuzestan. Interestingly, the majority (74.7%) of the G2a-P15 Y chromosomes belong to the paragroups G2a-P15* and G2a-P303* [49].

**Haplogroup E** in Iran is mainly represented by the E1-M123 (3.7%) and E1b-M78 (3.0%) branches. The first is almost entirely characterized by its sub-lineage M34 and reaches its highest incidence (13.6%) in Kurdistan. The second is present as E1b-M78* in Lorestan (9.8%) and E1b-V13 (5.9%) and E1b-V22 (2.9%) in the Zoroastrians of Yazd. It is worth noting the presence of individuals carrying African-specific haplogroups (three belonging to E2-M75 and 17 to E1b-M2) in South-East Iran (Hormozgan and Sistan Baluchestan), whereas the North-East African E1b-M81 is not observed.

### Phylogeography of the major Iranian haplogroups

**Figure 2:**
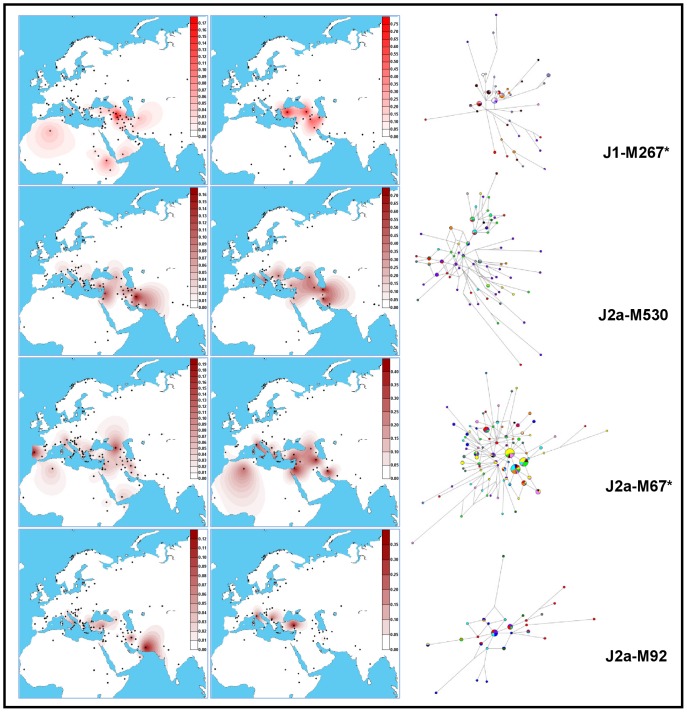
Frequency and variance distributions of haplogroup J lineages observed in Iran together with the relative networks of the associated STR haplotypes. Left panels: frequency distributions; central panels: variance distributions; right panels: networks. The areas of circles and sectors are proportional to the haplotype frequency in the haplogroup and in the geographic area, respectively, (for details about the colours, see Figures S2, S3, S4).

The main Iranian Y-chromosome haplogroups were further investigated for a set of microsatellites and the obtained results, together with data from literature (Tables S2, S3, S4, S5), were used to draw maps of variance and evaluate the age of their internal variation. Frequency and variance maps of the most informative haplogroups, together with the networks showing the relationships among their associated haplotypes (Table S6), are illustrated in figure 2. The age estimates per haplogroup per population/area are reported in Table S7.

#### Evidence of Late Glacial expansions from a Near Eastern Y-chromosome reservoir

It is known that in parts of the Near East, such as the Levant and Asia Minor, populations persisted throughout the last glaciation but no archaeological evidence for a Near Eastern Late Glacial expansions has till now been discovered. Recently, thanks to the recalibration of the mitochondrial DNA (mtDNA) clock [61], signals of Near Eastern dispersals towards Europe in the Late Glacial (from 12–19 kya) emerged from complete mitochondrial genome analysis of haplogroups J and T, previously associated only with the Neolithic diffusion [62]. Although the Y-chromosome molecular clock is far from reaching the mtDNA level of accuracy, evidences of Late Glacial dispersals from the Middle East are provided by the large number of deep rooting lineages (rare elsewhere), from which diverged different branches that underwent Neolithic expansions. Accordingly, Y chromosomes F-M89* and IJ-M429* were observed in the Iranian plateau: the first represents the ancestral state of the main Euro-Asiatic haplogroups [36] while the second probably moved toward southeast Europe sometime before the Last Glacial Maximum where it differentiated into the “western Eurasian” haplogroup I [27]. Similarly, basal lineages of the “Middle Eastern” haplogroup J (J1-M267* and different J2a lineages: J2-M172*, J2a-M410* and J2a-Page55*) and of haplogroups G (G2-P287*, G2-P15* and G2-P303*) and R (R1b-M269*) were also observed. Their frequency and variance distributions suggest a Mesolithic Middle Eastern origin/presence (Figure 1, Tables S2, S3, S4, S5 and S7) of these Y chromosomes supporting the role of the Middle East as a genetic reservoir for Late Glacial expansions and subsequent Neolithic dispersals southwards and westwards into South-East Europe.

J1-M267* shows high variance in the Middle Eastern region including Eastern Turkey, North-West Iraq [20], [43] and North-West Iran (Gilan – Mazandaran, Table S2), where probably originated 26.3±8.2 kya (Table S7) and then migrated westwards up to the Balkans and the Italian Peninsula and southwards as far as in Saudi Arabia and Ethiopia. The network of the M267* haplotypes (Figures 2 and S2) confirms the previously described non star-like substructure [43] enlightening a recent expansion (5.5±2.9 kya, Table S7) of the cluster characterized by the DYS388-13 and DYS390-23 repeats including North-East Turkish and Assyrian (from Turkey, Iraq and Iran) Y-chromosomes. This cluster harbours also virtually all the M267* Marsh Arab Y chromosomes supporting the previously proposed origin in northern Mesopotamia for the Iraqi Marsh Arabs [20]. However, only a further subdivision of this paragroup will allow a better understanding of times and ways of migrations marked by the M267* Y chromosomes.

Among the different J2a haplogroups, **J2a-M530** [46] is the most informative as for ancient dispersal events from the Iranian region. This lineage probably originated in Iran where it displays its highest frequency and variance in Yazd and Mazandaran (Figure 2). Taking into account its microsatellite variation and age estimates along its distribution area (Tables S3 and S7), it is likely that its diffusion could have been triggered by the Euroasiatic climatic amelioration after the Last Glacial Maximum and later increased by agriculture spread from Turkey and Caucasus towards southern Europe. The high variance observed in the Italian Peninsula is probably the result of stratifications of subsequent migrations and/or of the presence of sub-lineages not yet identified. Of interest in the M530 network (Figures 2 and S3) is the presence of a lateral branch that is characterized by a DYS391 repeat number equal to 9. Differently from previous observations [46], this branch is not restricted to Anatolian Greek samples being shared with different eastern Mediterranean coastal populations. The M530 diffusion pattern seems to be also shared by the paragroups J2a-M410* and J2a-PAGE55*. In addition, the variance distribution of the rare R1b-M269* Y chromosomes, displaying decreasing values from Iran, Anatolia and the western Black Sea coastal region, is also suggestive of a westward diffusion from the Iranian plateau, although more complex scenarios can be still envisioned because of its non-star like structure.

Another lineage potentially informative in revealing pre-Neolithic dispersals from the Middle East towards Europe is J2a-M67*. It is characterized by a wide distribution, including European, North-African and Near Eastern Y chromosomes, without virtually going beyond Afghanistan and Pakistan [24], [29], [63], [64]. Its variance distribution identifies different frequency peaks in Iran, the Levant, Cyprus, Crete and Central Italy (Figure 2). The network (Figures 2 and S4), which appears to be complex reflecting internal heterogeneity, includes three most frequent, one step related, haplotypes harbouring chromosomes from different populations, few common haplotypes (within population sub-sets) and a wide number of singleton haplotypes. Expansion events are clearly identified in the Levant and the Anatolia/Caucasus/southern Balkan regions from where the M67* spread towards southern Europe [41], [60]. Differently, no sign of J2a-M67* expansion is registered in other areas at high variance such as Iran (15.8±4.0 kya), Cyprus (14.8±4.0 kya), Central Italy (13.2±4.2 kya) and Crete (12.9±4.5 kya) (Table S7) where the majority of the observed haplotypes are rare and occupy a peripheral position in the network. Thus, while the high M67* variance in Central Italy is likely due to a stratification of seaborne migrations of Middle Eastern/Asia Minor peoples, the diversification observed in Iran and the Aegean Islands can be explained by a first Near Eastern, and possibly Anatolian, diffusion of the lineage followed by a Levantine expansion.

#### Haplogroup R1a and the diffusion of Indo-European languages

The diffusion of the Iranian branch of Indo-European languages whose origin is generally attributed to a western Asian region which includes Anatolia, the South Caucasus and the North Pontic-Caspian area [65], [66]; has been linked by numerous authors to the R1a haplogroup dispersal [8], [67], [68]. However, in spite of the recent dissection of this haplogroup, none of the identified sub-branches support a patrilineal gene flow from western Eurasia through southern Asia ascribable to the diffusion of Indo-European languages [45]. Accordingly, the present analysis of the Iranian R1a Y-chromosomes does not provide useful information to disentangle this issue. Indeed, the Iranian Y-chromosomes, as the majority of the European and virtually all the Asian ones, are still part of the unresolved paragroup R1a-M198* and harbour haplotypes shared by both European and Asian Y chromosomes.

#### Recent gene flows from neighbouring populations

Traces of recent gene flows from Arab countries and Anatolia are revealed in the Iranian Y-chromosome gene pool by the presence of the well-resolved sub-haplogroups J1-Page08 and J2-M92, respectively. The “Arab” **J1-Page08**, likely originated in the region at the border between south-eastern Turkey and North Iraq, underwent an important Neolithic expansion in the southern countries of the Middle East and represents the most important haplogroup in the modern populations of the Arabian Peninsula and North Africa [20], [43]. This lineage is observed at an averaged frequency of 6% in Iran, reaching a value in the Khuzestan Arabs (31.6%, Table 1), which is comparable to that observed in the neighbouring Iraqi population [20]. **J2a-M92** is a well-defined J2a-M67 sub-lineage, with a distribution restricted to Asia Minor, the Balkans and the north-eastern Mediterranean coasts. Frequency and variance maps make plausible an origin in north-western Turkey, where the highest variance is registered, and a subsequent migration to the Balkans and then to the Italian Peninsula. In Iran it is sporadically observed with the only exception of Sistan Baluchestan where it reaches an incidence of 12.5%. According to the age estimate (1.3±1.3 kya, Table S7) of the microsatellite variation associated to J2a-M92, its presence in Iran is ascribable to recent gene flow.

### The Iranian populations in the Near Eastern context

In order to test the genetic structure of the Iranian population and understand the relationships among the different Iranian ethnic groups in comparison with neighbouring Asian, European and African populations, the AMOVA and principal component analyses of Y-chromosome haplogroup frequencies were carried out at comparable levels of molecular resolution level (Table 1).

#### Principal component analysis (PCA)

**Figure 3:**
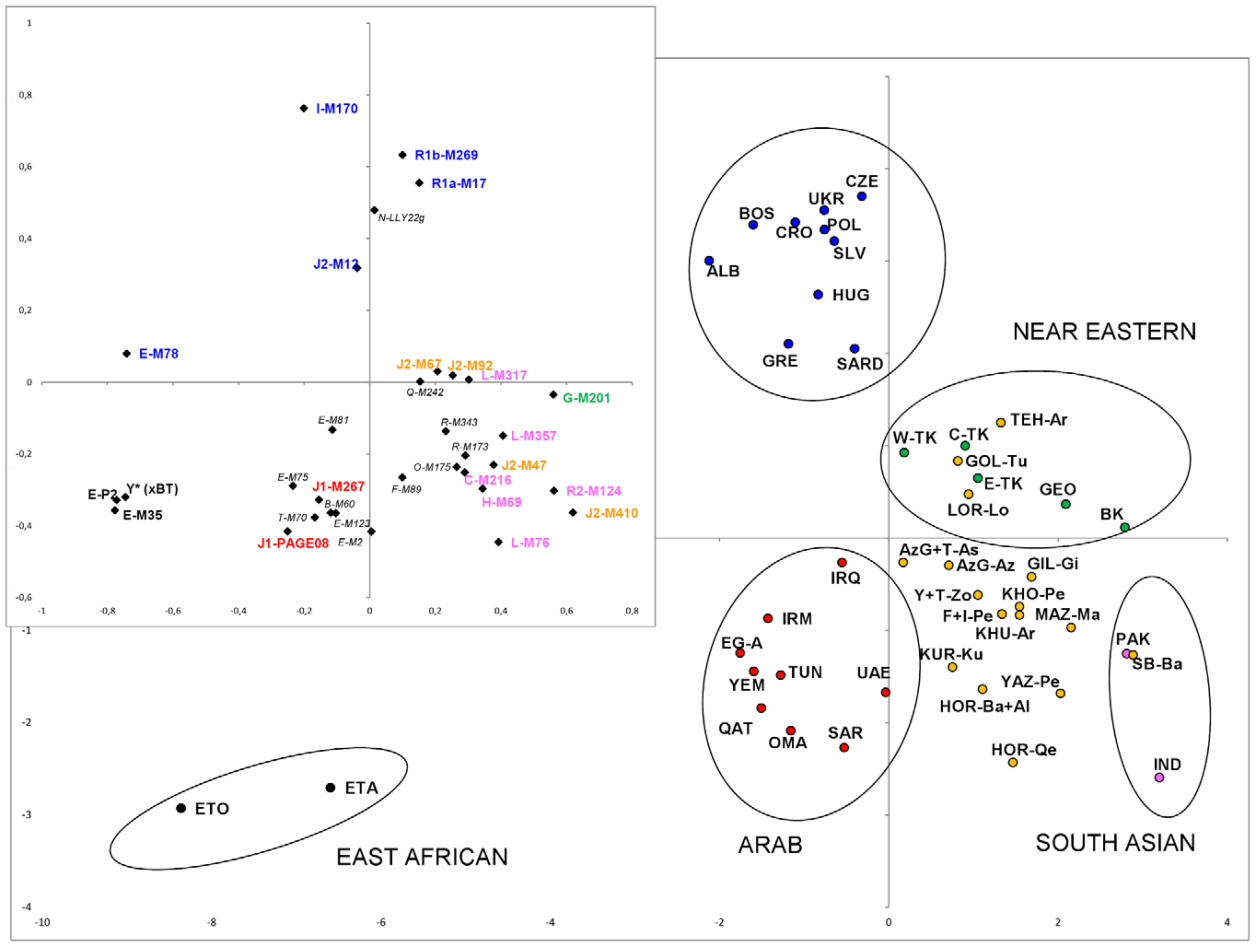
Principal component analysis performed using haplogroup frequencies in the Iranian populations of the present study (yellow) compared with those of relevant populations from the literature (East Africans in black, North African and Near Eastern Arabs in red, Europeans in blue, Turks and Caucasians in green and South Asians in pink). For population codes, see Table S1. On the whole, 25% of the total variance is represented: 14% by the first PC and 11% by the second PC. Insert illustrates the contribution of each haplogroup. Characterizing haplogroups are reported with the same population colours.

Although accounting only for 25% of the total variance, the first two components (Figure 3) separate populations according to their geographic and ethnic origin and define five main clusters: East-African, North-African and Near Eastern Arab, European, Near Eastern and South Asian. The 1^st^PC clearly distinguishes the East African groups (showing a high frequency of haplogroup E) from all the others which distribute longitudinally along the axis with a wide overlapping between European and Arab peoples and between Near Eastern and South Asian groups. The 2^nd^PC separates the North-African and Near Eastern Arabs (characterized by the highest frequency of haplogroup J1) from Europeans (characterized by haplogroups I, R1a and R1b) and the Near Easterners from the South Asians (due to the distribution of haplogroups G, R2 and L). Iranian groups do not cluster all together, occupying intermediate positions among Arab, Near Eastern and Asian clusters. In this scenario, it is worth of noticing the position of three Iranian groups: (i) Khuzestan Arabs (KHU-Ar) who, despite their Arabic origin, are close to the Iranian samples; (ii) Armenians from Tehran (THE-Ar), whose position, in the upper part of the Iranian distribution, indicates a close affinity with the Near Eastern cluster, while their position near Turkey and Caucasus groups, due to the high frequency R1b-M269 and other European markers (eg: I-M170), is in agreement with their Armenia origin; (iii) Sistan Baluchestan (SB-Ba) that clusters with its neighbouring Pakistan.

#### AMOVA analysis

Table 2 reports the results obtained by AMOVA macro- and micro-geographic tests performed adopting different grouping criteria (geographic, ethnic, linguistic and religious). As expected, before grouping, the majority of variability was observed within populations (84.69% for macro-geographic analysis and 96.45%, for micro-geographic analysis). After grouping, a great degree of geographic rather than linguistic correlation with the genetic structuring of the examined populations emerges, but the test was performed at lower resolution due to the necessity of making our data comparable with the published ones. Conversely, when the test is carried out only on the Iranian populations, at the high resolution level reached in this survey, linguistic seems to play a major role, explaining the highest percentage of variation among the Iranian groups (2.69% vs 2.18%, 2.03% and 1.06% for geography, ethnicity and religion, respectively). However, the variation among populations within groups decreases when Baluchs (living in the south-eastern region of the country) are separated by the other north-western Iranian language groups, underlining the importance of the geographic distance.

**Table 2:** AMOVA analysis.

(A) MACRO-GEOGRAPHIC LEVEL			
Subdivision criterion	Source of variation	Variance components	Percentage of variation
**NO GROUPING (1 group)**			
	Among populations	0.07175**	15.31
	Within populations	0.39698	84.69
**GEOGRAPHY (10 groups)** a			
	Among groups	0.28853**	9.27
	Among populations within groups	0.25443**	8.17
	Within populations	2.56989**	84.13
**LANGUAGE (15 groups)** b			
	Among groups	0.26757**	8.59
	Among populations within groups	0.27624**	8.87
	Within populations	2.56989**	82.53

(B) MICRO-GEOGRAPHIC LEVEL			
Subdivision criterion	Source of variation	Variance components	Percentage of variation
**NO GROUPING (1 group)**			
	Among populations	0.01695**	3.55
	Within populations	0.46104	96.45
**ETHNIC GROUP (14 groups)** c			
	Among groups	0.00969*	2.03
	Among populations within groups	0.00753**	1.57
	Within populations	0.46104**	96.40
**GEOGRAPHY (14 groups)** d			
	Among groups	0.01042*	2.18
	Among populations within groups	0.00687**	1.44
	Within populations	0.46104**	96.39
**LANGUAGE (8 groups)** e			
	Among groups	0.01290**	2.69
	Among populations within groups	0.00646**	1.34
	Within populations	0.46104**	95.97
**RELIGION (3 groups)** f			
	Among groups	0.00512	1.06
	Among populations within groups	0.01560**	3.24
	Within populations	0.46104**	95.70

(A) Macro-geographic level: data from Table S1. (B) Micro-geographic level: data from Table 1.

* p<0.05;

** p<0.01.

a **Geographic groups:** Africa, Balkans, Caucasus, Central Asia, Europe, Italy, Middle East, Saudi Arabia, South Asia, Turkey.

b **Linguistic groups:** Albanian, Armenian, Austro-Asiatic, Burushaski, Cushitic, Dravidian, Georgian, Greek, Indo-Iranian, Italic, Semitic, Slavic, Tibeto-Burman, Turkic, Uralic.

c **Ethnic groups:** Afro-Iranians+Bandari, Arabs, Armenians, Assyrians, Azeris, Baluchs, Qeshmi, Gilaks, Kurds, Lurs, Mazandarani, Persians, Turkmens, and Zoroastrians.

d **Geographic groups:** Azerbaijan Gharbi, Fars, Gilan, Golestan, Hormozgan, Isfahan, Khorasan, Khuzestan, Kurdistan, Lorestan, Mazandaran, Sistan Baluchestan, Tehran, Yazd.

e **Linguistic groups:** Altaic (Golestan), Arab (Khuzestan), Armenian (Tehran Armenians), Assyrian (Azerbaijan Gharbi+Tehran Assyrians), Baluch (Sistan Baluchestan), Caspian (Gilan+Mazandaran), NW Iranian branch (Azeri+Kurds), Persian (Fars+Hormozgan+Isfahan+Khorasan+Yazd+Tehran Zoroastrians), SW Iranian branch (Lorestan).

f **Religious groups:** Muslim, Zoroastrian and Christian.

### Conclusions

In order to visualize the relationships among Iranian groups and their neighbouring populations, the Y-chromosome haplogroups were defined at high resolution in 938 Iranian samples from 14 Iranian provinces and belonging to 15 different ethnic groups. The results were analyzed following phylogeographic and population genetics approaches.

Frequency and variance distributions of the main haplogroups together with the network analyses and age estimates were suggestive of pre-agricultural expansions from the Iranian plateau toward Europe via Caucasus/Turkey (J2-M410*, J2-PAGE55*, J2-M530, and R1b-M269*) as well as more recent movements into the Iranian region from Asia Minor/Caucasus (J1-M267*, J2-M92), Central Asia (Q-M25), southern Mesopotamia (J1-Page08) and from West Eurasia (R1b-L23 and probably part of R1a-M198*).

In brief, the Iranian gene pool has been at different times an important source of the Near Eastern and Eurasian Y-chromosome variability as well as a recipient of variation entered with different migratory events. The complexity of the Iranian male gene pool is well described by the PC analysis where some of the Iranian groups fall within the Near Eastern and South Asian clusters. Different factors could have contributed to the observed Iranian population heterogeneity, in particular the presence of important geographic barriers such as the Zagros and Alborz Mountain ranges and the two arid areas, the Dasht-e Kavir and the Dash-e Lut deserts. Both types of barriers, running from North-West to South-East, have limited gene flows from neighbouring regions and free movements of internal peoples, starting from the first peopling of this area. Their effects emerge from the distribution of all main Iranian Y-chromosome lineages and, in particular, from those of the two autochthonous Middle Eastern haplogroup J branches, J1-M267 and J2-M172 which display opposite distribution at the two sides of the Zagros Mountains, with the first prevalent in Iraq and Saudi Arabian Arab populations, and the second in the Iranian plateau, Anatolia and southern Europe. The Zagros Mountains represent a boundary also for the distributions of haplogroup R1a-M198. Although a further dissection of this Euro-Asiatic haplogroup is necessary to understand the population source of the Iranian R1a chromosomes, this haplogroup is less frequent in the western side of this mountain range. As for the distribution of haplogroup R1b-L23 (xM412), it is frequent in the north-western area of the country, whereas its incidence rapidly declines southwards from Lorestan. Differently, higher levels of heterogeneity are revealed in entrance or transit areas such as, for example, those observed in the populations living around the Caspian Sea, a situation that could be ascribed to population movements from and to Europe.

The overall scenario seems to indicate an autochthonous non-homogeneous ancient Y-chromosome gene pool, mainly composed by J2a sub-clades that was further shaped and enriched by the arrival of different populations during and after the Neolithic period. Western Eurasian contribution (mainly represented by R1b-L23, and at a lesser extent, by haplogroup sub-lineages I-M423 and J2-M241) is frequent in North-West Iran; Central Asian contribution (due to haplogroups H-M69, O-M175, Q-M242 and R2-M124) has its highest frequency in Khorasan, the easternmost province of the country. A clear African component is observed in Hormozgan where noteworthy is the presence of the sub-Saharan haplogroup E-M2 in the Afro-Iranian ethnic group.

In spite of the different geographic contributions and the presence of important geographic barriers which may have limited gene flows, AMOVA analysis revealed that language, more than geography, has played the main role in shaping the nowadays Iranian gene pool. Overall, the results of this study provide an accurate and reliable portrait of the Y-chromosomal variation in the modern Iranian populations, useful for generating a more comprehensive history of the peoples of this area as well as for reconstructing ancient migration routes. In addition, our results evidence the important role of the Iranian plateau as source and recipient of gene flows among culturally and genetically distinct populations.

### URLs

Encyclopaedia Britannica Online: http://www.britannica.com/

Fluxus Engineering: http://www.fluxus-technology.com

International Society Of Gene Genealogy: www.isogg.org

STR DNA Internet Data Base information: http://www.cstl.nist.gov/biotech/strbase/y20prim.htm

The Y chromosome Consortium: http://ycc.biosci.arizona.edu

## Supporting Information

Figure S1 **Phylogeny of Y-chromosome haplogroups observed the Iranian population.** The markers M33 and M81 of haplogroup E, M287, L91, and L497 of haplogroups G, M323 of haplogroup Q and M18, M434 and M458 of haplogroup R were typed but not observed. A star (*) indicates a paragroup: a group of Y chromosomes not defined by any reported phylogenetic downstream mutation.
(PDF)

Figure S2 J1-M267* reduced median network. The areas of circles and sectors are proportional to the haplotype frequency in the haplogroup and in the geographic area, respectively.
(TIF)

Figure S3 **J2-M530 reduced median network.** The areas of circles and sectors are proportional to the haplotype frequency in the haplogroup and in the geographic area, respectively.
(TIF)

Figure S4 **J2-M67 reduced median network.** The areas of circles and sectors are proportional to the haplotype frequency in the haplogroup and in the geographic area, respectively.
(TIF)

Table S1 Absolute frequencies of Y-chromosome haplogroups and subhaplogroups in the 44 populations included in the PCA.
(XLSX)

Table S2 Haplogroup J1-M267* frequencies and variances.
(XLSX)

Table S3 Haplogroup J2-M530 frequencies and variances.
(XLSX)

Table S4 Haplogroup J2-M67 frequencies and variances.
(XLSX)

Table S5 Haplogroup J2-M92 frequencies and variances.
(XLSX)

Table S6 Haplotypes used for age estimates and network constructions.
(XLSX)

Table S7 Age of microsatellite variation and Standard Error within the main haplogroups.
(XLS)
