# Supplementary figures and images for: Ancient Migratory Events in the Middle East: New Clues from the Y-Chromosome Variation of Modern Iranians
Source: PLoS One. 2012 Jul 18;7(7):e41252. doi: 10.1371/journal.pone.0041252 (PMC3399854; doi:10.1371/journal.pone.0041252)

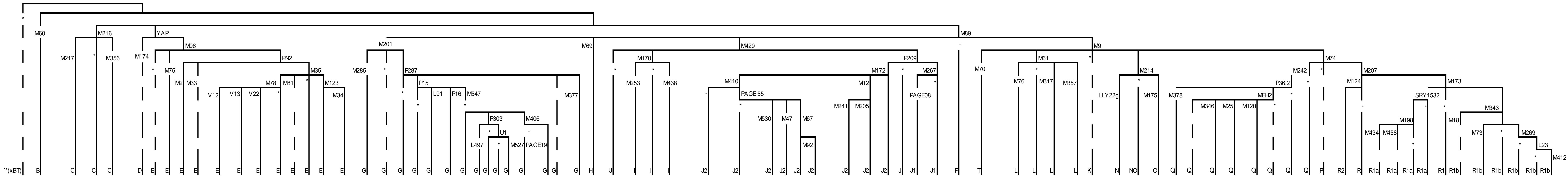

Supplement: Figure S1 — Phylogeny of Y-chromosome haplogroups observed the Iranian population. The markers M33 and M81 of haplogroup E, M287, L91, and L497 of haplogroups G, M323 of haplogroup Q and M18, M434 and M458 of haplogroup R were typed but not observed. A star (*) indicates a paragroup: a group of Y chromosomes not defined by any reported phylogenetic downstream mutation. (PDF) [file pone.0041252.s001.pdf]

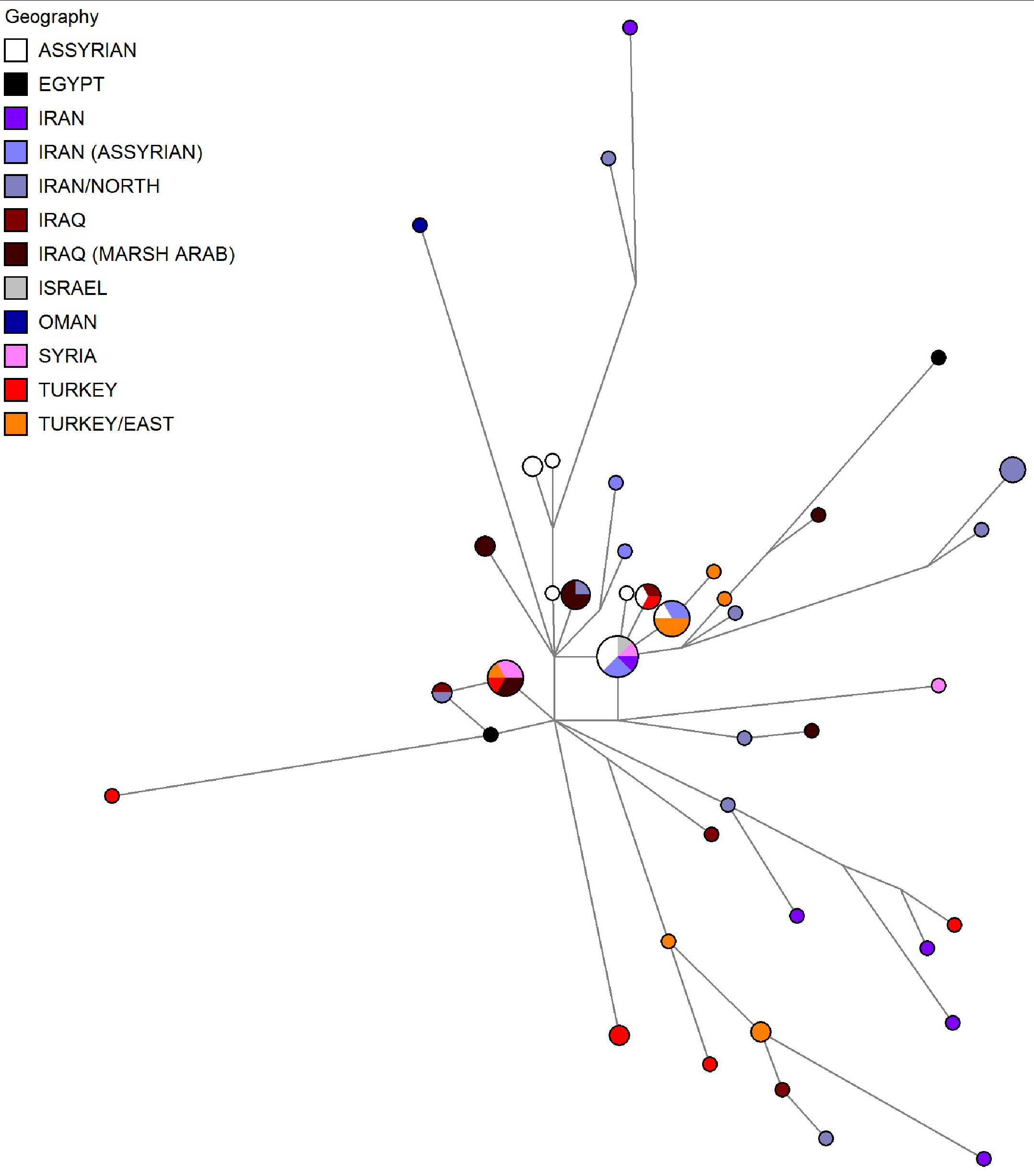

Supplement: Figure S2 — J1-M267* reduced median network. The areas of circles and sectors are proportional to the haplotype frequency in the haplogroup and in the geographic area, respectively. (TIF) [file pone.0041252.s002.tif]

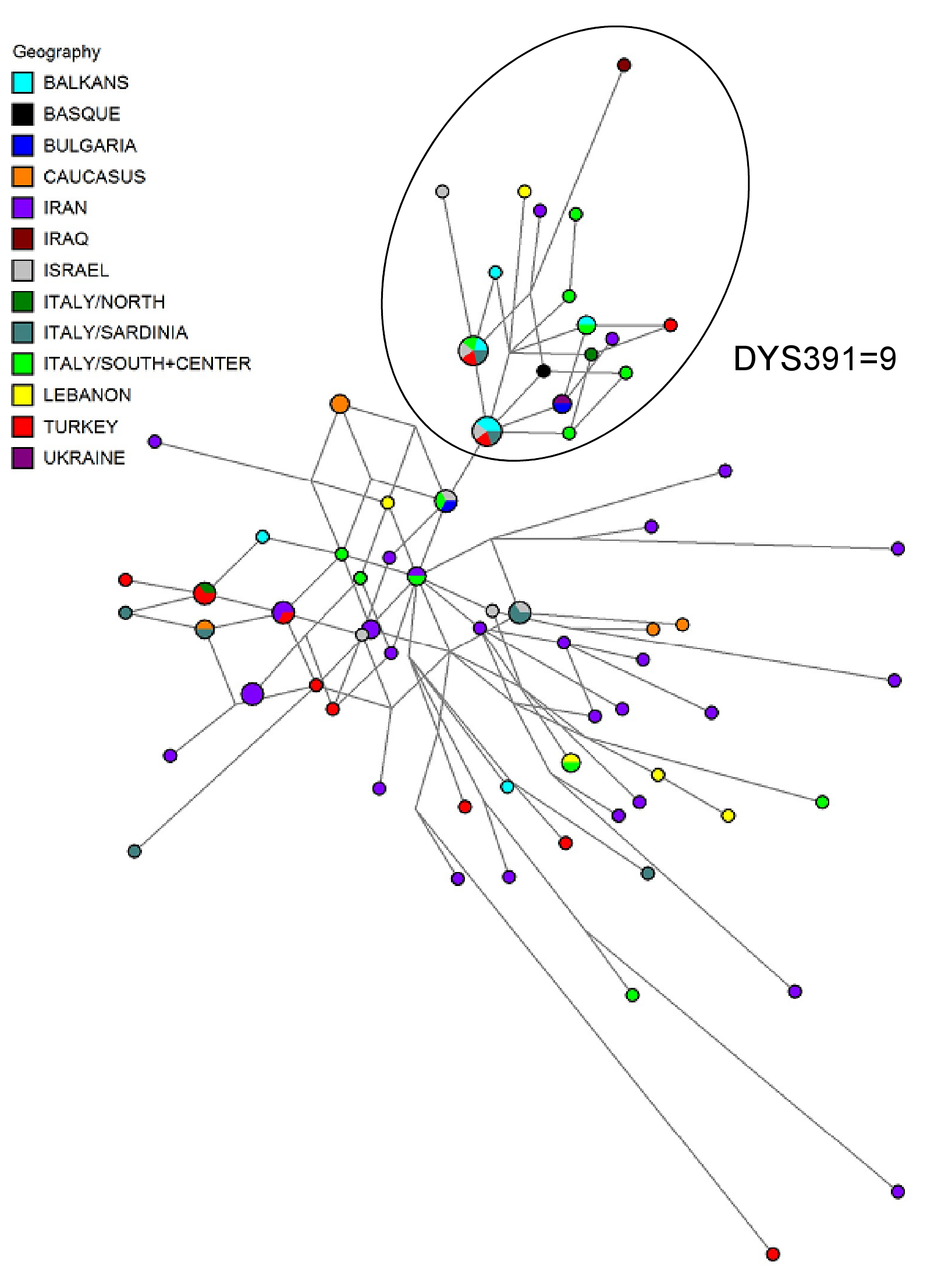

Supplement: Figure S3 — J2-M530 reduced median network. The areas of circles and sectors are proportional to the haplotype frequency in the haplogroup and in the geographic area, respectively. (TIF) [file pone.0041252.s003.tif]

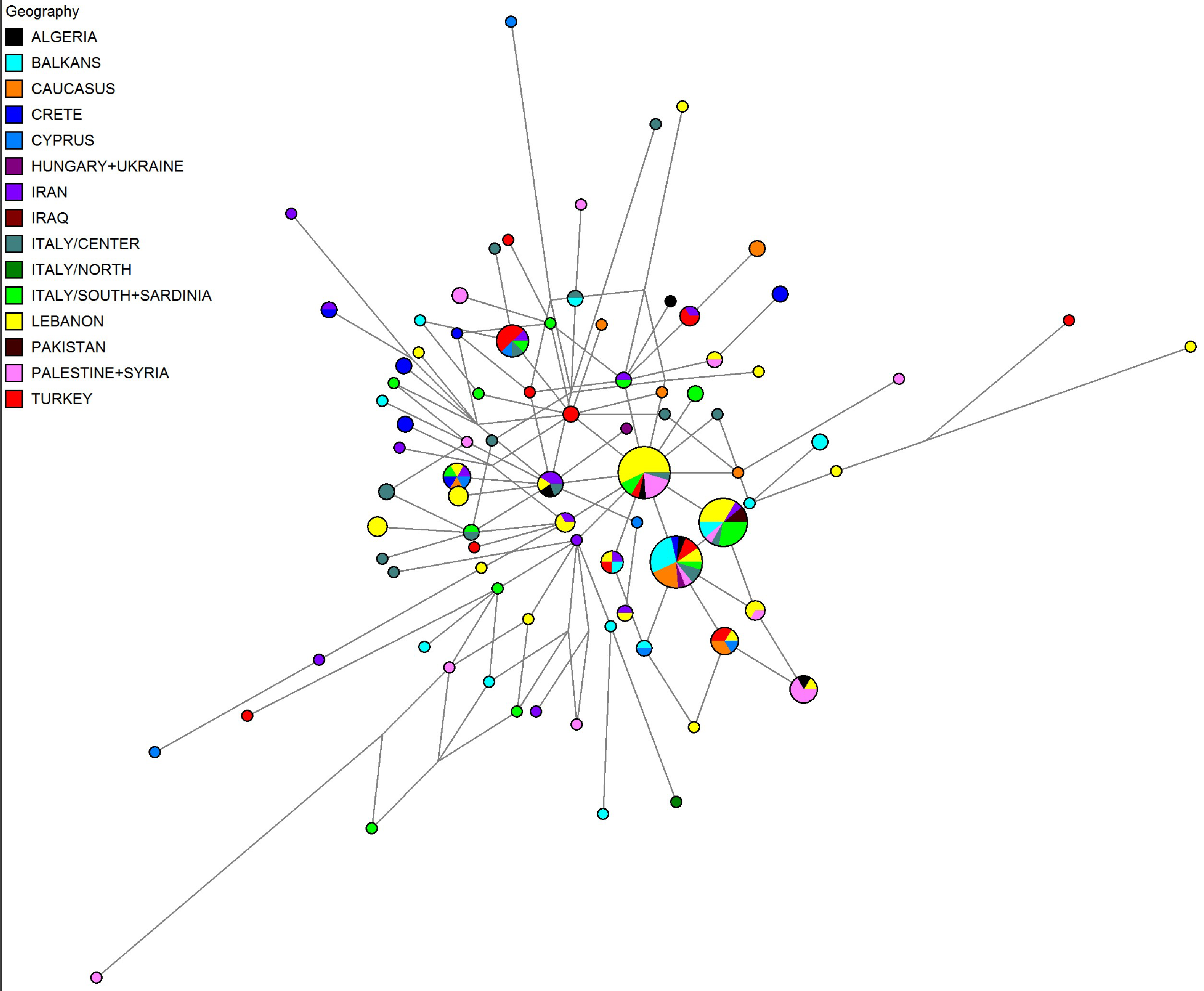

Supplement: Figure S4 — J2-M67 reduced median network. The areas of circles and sectors are proportional to the haplotype frequency in the haplogroup and in the geographic area, respectively. (TIF) [file pone.0041252.s004.tif]
